# Transvenous Radiofrequency Catheter Ablation for an Aldosterone-Producing Tumor of the Left Adrenal Gland: A First in Human Case Report

**DOI:** 10.1007/s00270-023-03584-x

**Published:** 2023-11-16

**Authors:** Sota Oguro, Hideki Ota, Satoru Yanagaki, Masahiro Kawabata, Hiroki Kamada, Kei Omata, Yuta Tezuka, Yoshikiyo Ono, Ryo Morimoto, Fumitoshi Satoh, Hiroaki Toyama, Kouta Tanimoto, Daisuke Konno, Masanori Yamauchi, Yuki Niwa, Hisao Miyamoto, Kenji Mori, Tetsuhiro Tanaka, Hiroshi Ishihata, Kei Takase

**Affiliations:** 1 Diagnostic Radiology, Tohoku University Graduate School of Medicine, 2-1 Seiryo-Machi, Aoba-ku, Sendai, Miyagi 980-8575 Japan; 2 Department of Diabetes, Metabolism and Endocrinology, Tohoku University Hospital, Sendai, Miyagi Japan; 3 Division of Nephrology, Rheumatology, and Endocrinology, Tohoku University, Sendai, Miyagi Japan; 4 Department of Anesthesiology, Tohoku University Graduate School of Medicine, Sendai, Miyagi Japan; 5 Research & Development Department, Japan Lifeline Co., Ltd., Saitama, Japan; 6 Department of Periodontology and Endodontology, Tohoku University Graduate School of Dentistry, Sendai, Miyagi Japan

**Keywords:** Transvenous catheter radiofrequency ablation, Aldosterone-producing adrenal tumor, First in human, Segmental adrenal vein sampling

## Abstract

**Purpose:**

To describe a novel technique of transvenous radiofrequency catheter ablation of an aldosterone-producing adenoma (APA) of the left adrenal gland using the GOS System (Japan Lifeline, Tokyo, Japan). Using the GOS system, a flexible radiofrequency tip catheter can be inserted into the adrenal central and tributary veins, the drainers for functional tumors.

**Materials and methods:**

An APA at the left adrenal gland, which was diagnosed by segmental adrenal venous sampling following administration of 0.25 mg cosyntropin, was ablated using the GOS catheter inserted into adrenal tributary veins via a right femoral vein 7-Fr sheath. The effect of radiofrequency ablation on APA was assessed using the international consensus on surgical outcomes for unilateral primary aldosteronism (PA).

**Results:**

No device-related complications were observed. The patient was deeply sedated under blood pressure and heart rate control with continuous administration of β-blockers. Then, the tumor and surrounding adrenal gland were cauterized at 7000 J two times each in sequence. The output time was 7−11 min for each ablation and 80 min in total. For blood pressure and pulse rate control, esmolol hydrochloride and phentolamine mesylate were used. The contrast enhancement of APA disappeared on dynamic CT immediately after the procedure. PA was biochemically cured until 12 months after the procedure.

**Conclusion:**

Using the radiofrequency device with the GOS catheter and system is a method for cauterizing adrenal tumors from blood vessels. This approach resulted in a marked reduction in aldosterone concentrations and a complete biochemical cure of PA over the observation period.

**Graphical Abstract:**

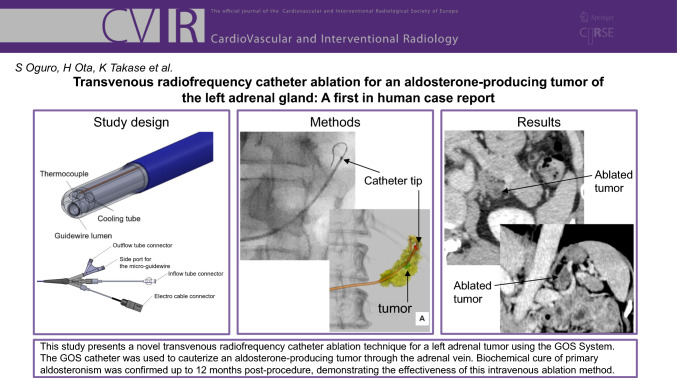

## Introduction

The most common subtypes of primary aldosteronism (PA) are aldosterone-producing adenomas (APAs) and bilateral adrenal hyperplasia. Laparoscopic adrenalectomy has been the standard treatment for benign APA, and most laparoscopic adrenalectomies for PA involve total removal of the affected adrenal gland. More recently, partial adrenalectomy has been performed to avoid postoperative adrenal insufficiency in multiple adrenal tumors [1, 2]. Additionally, percutaneous radiofrequency ablation (RFA) of APA under CT guidance has been applied [3]. CT-guided RFA can also preserve adjacent adrenal tissues and is reportedly effective [4]. However, in cases lacking a safe puncture route, especially in the left adrenal gland, percutaneous RFA is challenging to perform. Furthermore, when the tumor cannot be visualized on CT, partial adrenalectomy or CT-guided RFA is impossible. A novel approach to overcoming these challenges has been investigated in a recent study, focusing on a transvenous adrenal ablation technique using a flexible radiofrequency tip catheter. This method, tested in a swine model, allows for precise catheter placement into the adrenal central or tributary veins, eliminating the need for external puncture routes or CT visualization of the tumor [5]. The finding of the aforementioned preclinical study could be a promising therapeutic option. This system offers a tool for transvenous ablation of APA by allowing the insertion of a flexible radiofrequency tip catheter into the veins, draining functional adenomas. Herein, we present our first clinical procedure using the GOS system, focusing on its implementation and results and the use of segmental adrenal vein sampling (SAVS) to guide the accurate placement of the ablation catheter tip.

## Materials and Methods

The institutional review board approved this procedure. Written informed consent was obtained from the patient for the publication of the case details and any accompanying images.

### Case History and Physical Examination

**Fig. 1:**
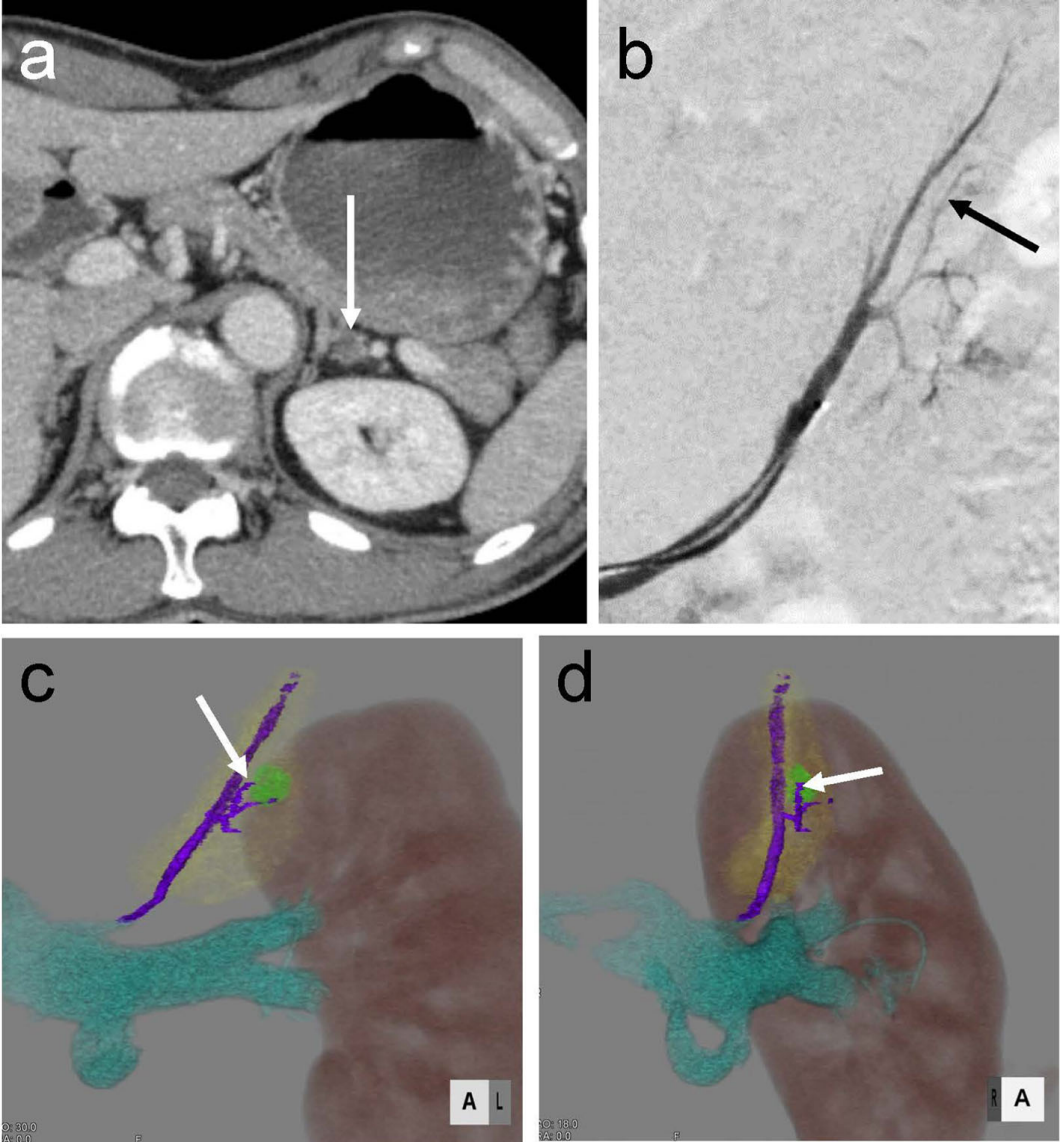
Left adrenal tumor in a 60-year-old man with primary hyperaldosteronism **a** Dynamic CT section through the left adrenal gland demonstrates a slightly enhancing nodule, 1 cm in diameter (arrow), in the left adrenal gland. **b** Digital subtraction of left adrenal venogram demonstrates the left adrenal and tributary veins. A capsular vein is filled, but the inferior phrenic vein is not due to the valve at its junction with the adrenal vein. A bent section can be confirmed on the central side of the superior lateral tributary vein (arrowhead). **c**, **d** Volume rendering (VR) image obtained on dynamic CT showing the main trunk of the adrenal vein and tributary veins (purple), the left renal vein (cyan), an adrenal tumor (green), the left adrenal gland (yellow), and the left kidney (brown). The left anterior and right anterior oblique views on the VR images show a superior lateral tributary vein located adjacent to the adrenal tumor (arrow)

A 65-year-old man diagnosed with hypertension for the past 20 years occasionally experienced palpitations. His recent blood pressure measurements ranged from 140/80 mmHg to 160/80–95 mmHg, and his heart rate was between 85 and 105 bpm. His blood pressure was controlled by amlodipine (10 mg daily) and doxazosin (2 mg daily). Multiphasic CT revealed a 1 cm left adrenal tumor (Fig. 1a).

### Laboratory Studies and Preparation

At baseline, plasma aldosterone and renin concentrations were 26.9 ng/dL and 0.71 pg/mL, respectively, and the serum potassium level was 3.0 mmol/L. The normal range of the aldosterone and renin is 3.1–35.4 ng/dL and 2.5—45.1 pg/mL, respectively. Captopril challenge, saline infusion, and dexamethasone suppression tests were performed. Positive results from all these tests led to the diagnosis of PA. Esaxerenone (MINNEBRO Tablets, Daiichi Sankyo Healthcare Co., Ltd.) was administered to eliminate the effects of aldosterone overproduction before RFA. Phenoxybenzamine or doxazosin was not administered preoperatively.

### Segmental Adrenal Vein Sampling Technique and Results

SAVS with adrenocorticotropic hormone stimulation confirmed unilateral aldosterone excess from the superior tributary vein of the left adrenal gland [6, 7]. From the SAVS, the lateralization index of the left adrenal central vein was 17.4 (Table 1). The details of the SAVS are provided in Fig. 1b. The relationship between the positions of the main trunk of the adrenal vein, tributary veins, and adrenal tumor was evaluated by dynamic CT (Fig. 1c, d).

**Table 1:** Central and segmental AVS

	A	C	A/C	SI	CSI
IVC	19.17	11.7	1.64	NA	NA
Right adrenal					
CV	398	718	0.55	61.37	0.34
RND1	94	551	0.17	47.10	0.10
RND2	223	574	0.39	49.06	0.24
RND3	92	396	0.23	33.85	0.14
Left adrenal					
CV	6555	678	9.67	57.95	5.90
LD	7868	631	12.47	53.93	7.60
LND1	140	517	0.27	44.29	0.17
LND2	423	572	0.74	48.89	0.45

A, aldosterone; A/C, a ratio of aldosterone-over-cortisol; AVS, adrenal vein sampling; C, cortisol; CSI, contralateral suppression index; CV, central vein; IVC, inferior vena cava; LD, drainer segment of the left adrenal; LND, non-drainer segment of the left adrenal; RND, non-drainer segment of the right adrenal; SI, selectivity index

### Evaluation

The effect of RFA on APA was assessed using the international consensus on surgical outcomes for unilateral PA [8]. The severity of adverse events was assessed using the Common Terminology Criteria for Adverse Events, version 5.0 [9].

### Transvenous Radiofrequency Catheter Ablation of the Adrenal Adenoma and Results

**Fig. 2:**
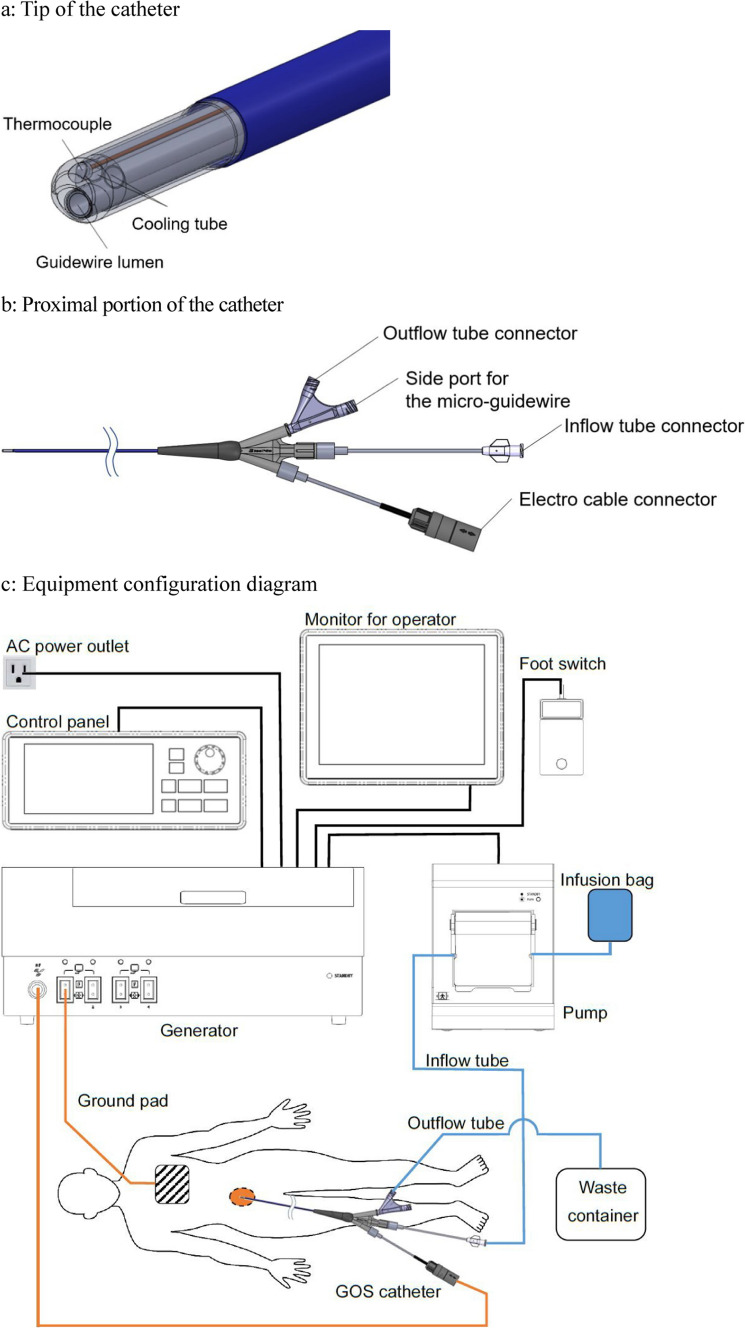
GOS system (Japan Lifeline Co., Ltd, Tokyo, Japan). **a** The GOS catheter has an original spiral-slit cut and is sufficiently flexible to follow the curvature of the venous vasculature. It was designed for a 6-Fr sheath, with a 1.65 mm shaft diameter and a guidewire lumen compatible with a 0.016 inch micro-guidewire. The estimated cauterization area with the catheter is approximately 18 mm in the longitudinal direction and 17 mm in the short-axis direction. **b** The catheter also has an inner cooling mechanism. **c** The ablation was performed using a 500-kHz radiofrequency generator. A ground pad was attached to the right back. The roller pump was capable of perfusing saline at 20 mL/min in the inner cooling mechanism of the catheter

**Fig. 3:**
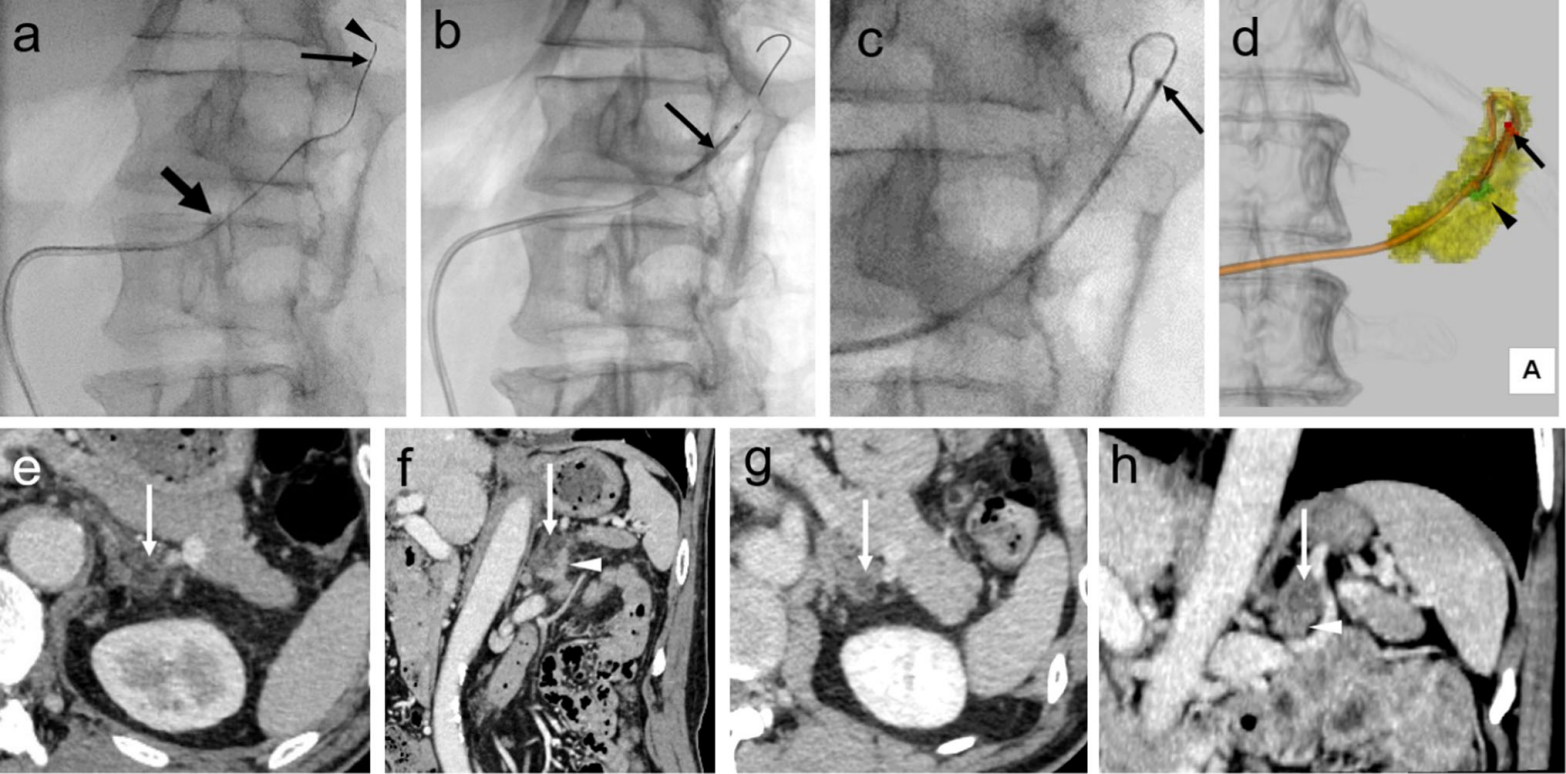
Transvenous catheter radiofrequency ablation of an aldosterone-producing adenoma of the left adrenal gland **a** The superior lateral tributary vein was catheterized using a 7-Fr guiding catheter (large arrow) inserted into the left adrenal vein, a 1.7-Fr microcatheter (arrow), and a 0.016 inch microwire (arrowhead). **b** The flexure part of the adrenal tributary vein is dilated using a 2.5 mm diameter, 1.5 cm length balloon catheter (Ikazuchi, KANEKA medical products, Tokyo, Japan) to provide a lumen for insertion of the GOS catheter (arrow). **c** The GOS catheter was advanced to the end of the superior lateral adrenal tributary vein over the wire (arrow). Then, the catheter tip was pulled back approximately 4 mm by each cauterization, and a total of four cauterizations were performed. The relationship between the catheter tip and the adenoma was not clear in the images, but it was possible to ascertain their relative positions to some extent by comparing the plain CT with the preoperative CT. **d** Volume rendering image obtained from non-enhanced CT images showing the GOS catheter (orange), tip of the catheter (red) (arrow), adrenal tumor (green) (arrowhead), and adrenal gland (yellow), providing a good view of the relationship between the tumor and catheter tip. **e**, **f** After system removal, dynamic CT using 60 mL of iodine-contrast medium was performed. On axial and coronal dynamic CT images, the left adrenal lesion is not detected as an enhanced tumor (arrow). Some edematous changes are seen around the adrenal gland. The lower part of the adrenal gland shows normal enhancement (arrowhead). **g**, **h** Five months after the procedure, a dynamic CT for screening of coronal arterial stenosis was performed. While the lower part of the adrenal gland shows enhancement (arrowhead), the left adrenal lesion did not show contrast enhancement (arrow)

The intervention was performed on an ANGIO-CT system. A radiofrequency tip catheter, the GOS catheter, was used for this procedure. The GOS catheter (Japan Lifeline Co., Ltd, Tokyo, Japan) is made of a stainless steel tube with an insulating coating (Fig. 2) [5]. First, a 7-Fr sheath was inserted at the right femoral vein by ultrasound guidance, and the left adrenal vein was catheterized using a 7-Fr guiding catheter (CX Guiding Catheter for the left adrenal vein, Large, Hanaco Medical, Tokyo, Japan). Then, a 1.7-Fr microcatheter was advanced into a tributary vein adjacent to the APA (Fig. 3a), and a 0.014-inch and 300 cm micro-guidewire (ThruwayTM Boston Scientific Co. Massachusetts) was inserted into the tributary vein. There was a bent section of the adrenal vein (Fig. 1b). Initially, we attempted to advance the GOS catheter into the adrenal tributary vein, but due to the bend in the adrenal tributary veins, it was impossible to gently advance the GOS catheter into it. Consequently, we determined that dilating the bent section using a 2.5-mm wide and 1.5-cm-long balloon catheter (Ikazuchi, KANEKA medical products, Tokyo, Japan) was preferable to forcefully advancing the GOS system, which could have resulted in damage to the tributary veins. Finally, the GOS catheter was advanced into it (Fig. 3b, c). After confirming the location of the tip of the GOS catheter on plain CT (Fig. 3d), the 0.014-inch micro-guidewire was removed, and saline was infused into the guidewire lumen at 30 mL/h. Heparin was not used in this procedure.

The patient was deeply sedated under blood pressure and heart rate control with continuous administration of β-blockers and a radial arterial catheter placed to monitor blood pressure. Then, the APA and surrounding adrenal gland were cauterized at 7000 J two times each in sequence. In the catheter tip used in this report, thermocouples were placed inside the electrodes, allowing the electrode temperature to be measured. The temperature at the tip was measured, while the ablation was performed. The output time was 7−11 min for each ablation and 80 min in total. For blood pressure and pulse rate control, 1308 mg of esmolol hydrochloride (Brevibloc, Maruishi Pharmaceutical. Co., Ltd, Japan) was continuously administered, a total of 190 mg of esmolol hydrochloride as bolus injections, and 32 mg of phentolamine mesylate (Regitin injection, Novartis Pharma K.K., Tokyo, Japan). For pain, 100 mg of tramadol hydrochloride (Tramal injection, Nippon Shinyaku Co., Ltd. Japan) and 1000 mg of acetaminophen (Acelio bag for intravenous injection, Terumo Corp. Tokyo, Japan) were administered.

## Results

Contrast enhancement of the APA disappeared on dynamic CT (Fig. 3e, f). The procedure took 4 h and 31 min, from the first local anesthesia to remove all sheaths. The maximum heart rate was 108 bpm, and the maximum systolic invasive and non-invasive blood pressures were 183 mmHg and 160 mmHg, respectively (grade 3). Radiation exposure for CT was a dose-length product of 1348 (mGy cm) and calculated effective dose of 20.2 mSv, and for fluoroscopy, a dose-area product of 35.16 (Gy*cm^2^) and calculated effective dose of 7 mSv, respectively [10]. No device-related complications were observed. Postoperatively, the patient was discharged on day 7, and the total duration of hospitalization was 15 days. Plasma aldosterone and renin concentrations decreased to 0.4 ng/dL and 0.55 pg/mL, respectively, and the serum potassium level was normalized at 12 months. Post-procedural dynamic CT scans were scheduled for 6 and 12 months, but there was a single asthma attack that occurred before the scheduled CT scans. Due to concerns over the potential adverse reactions of the contrast medium in the patient with a recent asthma attack, it was deemed safer to not administer contrast for both the 6- and 12-month CT scans. Hence, both scans were unenhanced. However, it was possible to evaluate the adrenal gland with the cardiac CT scan taken 5 months after the procedure, as it fell within the scan range. The adrenal adenoma showed a disappearance of the enhancement effect, while an enhancement effect was observed in the substance of the lower pole of the adrenal gland. One year after the surgery, the blood pressure and pulse rates were as follows: systolic blood pressure, 108 and 107 mmHg; diastolic blood pressure, 72 and 74 mmHg; and pulse rate, 78 and 79 bpm. PA was partially cured clinically and completely cured biochemically throughout the observation period, which was evaluated by PA surgical outcome criteria [8].

## Discussion

Transvenous radiofrequency catheter ablation was feasible for treating the left adrenal APA. The advantage of transvenous RFA is that it can be performed on a specific tributary vein exhibiting hypersecretion, as identified through SAVS. Consequently, the success rate of SAVS becomes vital. While the success rate of SAVS is reportedly as high as 98%, demonstrating a remarkably high level of success, there exists a procedural learning curve that may affect outcomes [6, 11].

One problem with APAs is the presence of nonfunctioning adenomas, which are difficult to distinguish from functioning tumors on CT or MRI. Partial adrenalectomy has become an alternative strategy, but it may fail symptom remission if nonfunctional adenomas are removed and microadenomas remain. In this context, the GOS system presents a significant advantage over surgery, as it can treat the responsible tributary vein adjacent to the lesion that was clinically proven to be APA.

The development and advancement of the GOS system were previously reported, demonstrating its capability to ablate surrounding lesions through intravascular insertion. Our preclinical animal study supported its effectiveness. However, complexities, such as the need for venous dilation, multiple ablation sessions, and difficulties in catheter insertion, were encountered, leading to an extended ablation time.

In the present study, the disappearance of the enhancing effect in the adrenal adenoma was revealed by postoperative contrast-enhanced CT, and biological remission was achieved 1 year postoperatively. Despite the challenges faced, the success in ablation highlights the potential of the GOS system as an innovative solution for adrenal adenoma treatment. Careful handling and precise control were essential.

The right central adrenal vein’s short length makes the insertion of a catheter a challenging procedure, requiring possible design improvements for future treatments. Additionally, there are potential improvement and optimization areas, including risks related to rupture or spasm during the procedure. Future research should aim at refining the technique, and further validation of its efficacy and safety in a broader spectrum of cases is warranted.

As for the necessity of CT verification for the proper positioning of the tip of the probe, cone beam CT could also be used. We could not find any study evaluating the radiation dose during percutaneous ablation therapy for PA. However, the radiation dose in this study was within the range of the reported dynamic abdominal CT study and adrenal venous sampling procedure [10, 12]. Furthermore, based on previous experience with RFA in other cases, it was judged that the procedure could be performed under sedation.

The current report includes several aspects, such as the number of ablations required for complete treatment, and still needs to be optimized. It is also essential to acknowledge that much of the discussion on the potential advantages of the RFA catheter (the GOS system) remains speculative at this stage.

We also noted the challenge of transvenous ablation for the right adrenal gland with current catheters, underscoring the need for improvements in future devices. Experimental evidence, including measurements contributing to long-term blood pressure improvements and the evaluation taken a year later, further support the study’s findings.

## Conclusions

Transvenous radiofrequency catheter ablation seems to be a feasible alternative to partial adrenalectomy in patients with a left adrenal aldosterone-producing adenoma. The use of the radiofrequency device with the GOS catheter and system appears to be a safe method for the cauterization of adrenal tumors in this single-case experience and led to a marked reduction in aldosterone concentrations and a complete biochemical cure of PA over the observation period.
